# The Health-Sustaining, Moderating, and Mediating Roles of Sense of Coherence in the Relationship between Fear of COVID-19 and Burnout among South African Teachers

**DOI:** 10.3390/ijerph19095160

**Published:** 2022-04-24

**Authors:** Anita Padmanabhanunni, Tyrone Brian Pretorius, Ashraf Kagee

**Affiliations:** 1 Department of Psychology, University of the Western Cape, Bellville 7530, South Africa; tpretorius@uwc.ac.za; 2 Department of Psychology, Stellenbosch University, Stellenbosch 7600, South Africa; skagee@sun.ac.za

**Keywords:** manageability, meaningfulness, comprehensibility, personal accomplishment, emotional exhaustion, depersonalization, fear of COVID-19

## Abstract

The current study focuses on the interrelationship between fear of COVID-19, sense of coherence, and burnout. Participants (*n* = 355) were school teachers from across all provinces in South Africa who completed the Fear of COVID-19 Scale, the Sense of Coherence Scale, and the Maslach Burnout Inventory. It was hypothesized that the dimensions of sense of coherence would be directly associated with burnout and would also mediate or moderate the relationship between fear of COVID-19 and burnout. The results of the path and moderation analyses conducted confirmed this hypothesis. In particular, the health-sustaining role of sense of coherence was demonstrated through the significant direct associations between comprehensibility and manageability on one hand and emotional exhaustion, as well as depersonalization, on the other hand. In addition, meaningfulness had significant direct associations with emotional exhaustion, depersonalization, and personal accomplishment. Meaningfulness mediated the relationship between fear of COVID-19 and all burnout subscales, while comprehensibility and manageability only mediated the relationship between fear of COVID-19 and both emotional exhaustion and depersonalization. However, comprehensibility and manageability played a moderating role in the relationship between fear of COVID-19 and personal accomplishment. These findings confirm the crucial role of protective factors, such as sense of coherence, and highlights the need for interventions that could strengthen these resources within teachers.

## 1. Introduction

The COVID-19 pandemic and the measures taken to curb the spread of the SARS-CoV-2 virus have resulted in widespread fear of infection and triggered an unprecedented health crisis, especially for those at the frontlines of the service sector. Teachers represent a subgroup of this population that serves at the frontlines of the educational system. They have been impacted by the pandemic in unique ways. The pandemic has placed additional stresses on teachers, considering their vulnerability to infection in the workplace and the adaptations that needed to be made in their daily routines even though evidence has suggested that children are less likely to transmit the SARS-CoV-2 virus to adults [1]. Nonetheless, children have experienced disruptions in their learning, challenges to their mental and physical health, and poor nutrition as a result of COVID-19-related disruptions [2], which in turn have exacerbated the stress experienced by teachers. During the height of the pandemic, teachers worked from home even though some had limited to no online access. With the resumption of conventional classroom teaching, teachers were required to teach longer hours, prepare additional assessments, and work more days as the school year was extended in December 2020 [3].

The current study was conducted in South Africa, where school closures constituted some of the measures taken to curb the spread of the virus. Considerable uncertainty ensued when schools were reopened and then closed in June 2020 [3]. The already substantial workloads of teachers were further exacerbated by the need to switch to online delivery in addition to personal difficulties they encountered in managing the boundary between school and home [4]. Having family members in their new professional space, including children requiring care, created additional stressors. Teachers also faced similar challenges to other members of society, including concerns about their health and that of their loved ones, social distancing, restrictions on travel and movement, limited access to daily necessities, and uncertainty about the resumption of their “normal” lives [4]. In an international online survey of 634 language teachers, elevated levels of stress were reported. Positive psychosocial experiences such as good health, emotional fulfillment, personal growth, and resilience were found to correlate positively with approach coping and negatively with avoidant coping. Negative outcomes such as symptoms of anxiety, psychosocial stress, social isolation, and anger were associated with avoidant coping [4].

In South Africa, between mid-February to the end of April 2021, 171 excess teacher deaths were recorded. Nevertheless, over the December–January holidays, which corresponded to the second wave of COVID-19 infections, there were 1123 excess teacher deaths. Of the 401,327 teachers in the school system, 2283 were recorded to have died due to COVID-19 between the end of March 2020 and the end of May 2021, representing under 0.6% of the entire cohort of teachers [5]. Thus, the periods when schools were open and the observed excess deaths were not observed to be associated.

It has become apparent that children have not been the main drivers of the COVID-19 pandemic [1]. However, the sheer number of interpersonal interactions within a school over time places teachers at elevated risk of contracting COVID-19, from either learners or other staff members. Nonetheless, efforts to reduce the rate of infections have brought into focus the logistical complexities associated with physical distancing at school and within the classroom. These complexities include monitoring the use of masks and thermometers and managing at-risk teachers who may demand the right to stay at home [6].

Digital inequality has also become a contributing factor to stress and burnout. Learners from wealthy homes have greater access to the Internet than those from poorer homes, thus creating ethical dilemmas for teachers. Online teaching places learners who have limited or no Internet access at a disadvantage, which then needs to be mitigated by teachers [6]. As argued by [7], limited Internet access is a barrier to a digital literacy environment in the learning arena. Even when teachers themselves have digital access, they have reported experiencing challenges in learning to operate the new technology themselves. Teachers have had to adapt to digital teaching during times of school closure by seeking solutions to technological problems and obtaining advice from other educators [8].

Reports from outside South Africa have also documented challenges experienced by the teaching community. Indonesian teachers, for example, expressed concerns that when working from home they risked losing motivation to work and that they incurred greater electricity and Internet costs and data security threats [9]. For 1626 Canadian teachers, both their level of efficacy and how they perceived change and administrative support were associated with resilience and burnout at the onset of the pandemic. At the beginning of the pandemic, teachers reported experiencing increasing exhaustion and cynicism although efficacy in managing the classroom and their sense of accomplishment also increased, presumably due to increased training and experience with online teaching [10]. Among teachers in low- and middle-income countries, the transition to online learning environments was reported to create considerable stress and frustration for teachers [11]. In Portugal, it was found that teachers’ perceptions of their well-being decreased as a result of COVID-19, and some expressed concern about their professional future [12]. Among Palestinian teachers, limited Internet access was a signal of an equity crisis that Palestinian schools had already been experiencing for a prolonged period [13]. Turkish teachers similarly reported that students’ technical challenges, Internet connectivity problems, limited motivation to learn, the inability of parents to create an adequate learning environment, as well as their limited ability to support their children, were additional sources of stress [14].

People are unlikely to be equally affected by the stressors associated with the pandemic. Some may be more vulnerable to emotional and psychological distress than others. This suggests that protective factors that influence vulnerability to mental health outcomes may play a critical role in their responses to stressors. The current study focuses on one such protective factor—namely, sense of coherence (SOC)—and examines the various roles that it plays in shaping the relationship between fear of COVID-19 and burnout. SOC is a global orientation that reflects the extent to which individuals are able to perceive their environment as comprehensible, meaningful and manageable [15]. Comprehensibility refers to the belief that things that happen are rational and understandable, meaningfulness describes the belief that things that happen in life are meaningful and worthwhile, and manageability refers to the belief that resources are available to cope with adverse events [15]. SOC enables people to make use of adaptive coping strategies and in this way buffers against the negative effects of stress. Existing studies (e.g., [16]) have reported that SOC can serve as a protective factor against burnout. Burnout is a psychological syndrome arising from chronic exposure to work-related stress and is characterized by physical and emotional exhaustion, feelings of cynicism, a sense of detachment from the job and appraisals of being ineffective at one’s work [17].

First, it is presumed that SOC has a direct effect (health-sustaining) on indices of burnout. Accordingly, the following hypotheses are examined:

**Hypothesis** **1** **(H1).** *Comprehensibility, as a dimension of SOC, is negatively associated with emotional exhaustion.*

**Hypothesis** **2** **(H2).** *Comprehensibility is negatively associated with depersonalization.*

**Hypothesis** **3** **(H3).** *Comprehensibility is positively associated with personal accomplishment.*

**Hypothesis** **4** **(H4).** *Manageability, as a dimension of SOC, is negatively associated with emotional exhaustion.*

**Hypothesis** **5** **(H5).** *Manageability is negatively associated with depersonalization.*

**Hypothesis** **6** **(H6).** *Manageability is positively associated with personal accomplishment.*

**Hypothesis** **7** **(H7).** *Meaningfulness, as a dimension of SOC, is negatively associated with emotional exhaustion.*

**Hypothesis** **8** **(H8).** *Meaningfulness is negatively associated with depersonalization.*

**Hypothesis** **9** **(H9).** *Meaningfulness is positively associated with personal accomplishment.*

In addition, it is postulated that dimensions of SOC play an intermediary role (moderating or mediating) in the relationship between fear of COVID-19 and burnout. In this regard, the following hypotheses are postulated:

**Hypothesis** **10** **(H10).** *Comprehensibility mediates or moderates the relationship between fear of COVID-19 and emotional exhaustion.*

**Hypothesis** **11** **(H11).** *Manageability mediates or moderates the relationship between fear of COVID-19 and emotional exhaustion.*

**Hypothesis** **12** **(H12).** *Meaningfulness mediates or moderates the relationship between fear of COVID-19 and emotional exhaustion.*

**Hypothesis** **13** **(H13).** *Comprehensibility mediates or moderates the relationship between fear of COVID-19 and depersonalization.*

**Hypothesis** **14** **(H14).** *Manageability mediates or moderates the relationship between fear of COVID-19 and depersonalization.*

**Hypothesis** **15** **(H15).** *Meaningfulness mediates or moderates the relationship between fear of COVID-19 and depersonalization.*

**Hypothesis** **16** **(H16).** *Comprehensibility mediates or moderates the relationship between fear of COVID-19 and personal accomplishment.*

**Hypothesis** **17** **(H17).** *Manageability mediates or moderates the relationship between fear of COVID-19 and personal accomplishment.*

**Hypothesis** **18** **(H18).** *Meaningfulness mediates or moderates the relationship between fear of COVID-19 and personal accomplishment.*

## 2. Materials and Methods

### 2.1. Participants

Participants (*n* = 355) were school teachers recruited from all provinces in South Africa. The majority of the participants, however, were based in the Western Cape Province (82.3%). The Western Cape is ranked fourth in terms of number of teachers per province [17]. The mean age of the participants was 41.9 (SD = 12.42). Participants in the sample were mostly women (76.6%), worked in urban settings (61.7%), and taught at the primary school level (61.1%). South Africa had about 444,000 teachers in 2019 [18]; thus, the current sample contains a 5.09% margin of error (confidence interval of 95%).

### 2.2. Instruments

Participants in this study completed a brief demographic questionnaire. In addition, they also completed the Fear of COVID-19 Scale (FCV-19S; [19]), the short form of the Sense of Coherence Scale (SOC-13; [15]), and the Maslach Burnout Inventory (MBI; [17]).

The FCV-19S was developed to assess reactions of fear related to COVID-19. It consists of seven items measured on a 5-point Likert scale ranging from Strongly Disagree (1) to Strongly Agree (5). Ahurso and colleagues [19] demonstrated the reliability and validity of the FCV-19S using both classical test theory as well as Rasch analysis. The scale has also demonstrated strong psychometric properties in a range of contexts (e.g., Israel: [20], 2020; Turkey: [21]). The reliability, validity, and unidimensionality of the scale when used with South African teachers have also been demonstrated [22].

The SOC-13 measures a global orientation to life and entails viewing the world as comprehensible, meaningful, and manageable [15]. It consists of 13 items that measure these three dimensions of the construct “sense of coherence.” Participants responded to the items on a 7-point scale. Two systematic reviews [23,24] found that the scale had been used in 20 and 32 countries, respectively, and overall, the scale demonstrated sound reliability, validity, and cross-cultural applicability.

The MBI is the most extensive measure of burnout. It does not provide a global measure of burnout, but instead measures three separate dimensions reported to reflect burnout. The emotional exhaustion subscale refers to feelings of tiredness, fatigue, and being emotionally drained as a result of work. The depersonalization subscale refers to negative and cynical feelings toward students and colleagues. The personal accomplishment subscale refers to a sense of effectiveness and achievement with regard to work. Participants responded to the 22 items of the MBI using a 7-point Likert scale that ranged from 0 (Never) to 6 (Every day). While there has been a debate about the factor structure of the MBI, there has also been strong support for the proposed three-factor structure in different contexts (e.g., Colombia [25]; Spain [26]). In addition, Schaufelli and colleagues [27] confirmed the validity of the three-factor structure of the MBI and found that the emotional exhaustion and depersonalization dimensions were able to distinguish between burned out and non-burned out workers. The three-factor structure and the reliability of the MBI were also confirmed when used in an educational setting in South Africa [28].

### 2.3. Procedures

The Google Forms application was used to create an online version of all the instruments. Administrators from teacher Facebook groups were approached for permission to recruit participants via their sites. In addition, officials from the education department were briefed on the aims and objectives of the study and circulated the link to their colleagues.

### 2.4. Ethics

Ethical approval for the study was received from the Humanities and Social Sciences Ethics Committee of the University of the Western Cape (ethics reference number: HS21/3/8). The surveys were completed anonymously. Participants also had to provide informed consent on the first page of the online survey before they were allowed to proceed to the survey proper.

### 2.5. Data Analysis

Descriptive statistics, reliabilities (alpha and omega), and the intercorrelations between variables were obtained using IBM SPSS Statistics for Windows (version 26, IBM Corp., Armonk, New York, USA. To obtain the omega coefficient, the OMEGA macro [29] for SPSS was used. The direct effects of fear of COVID-19 and the SOC subscales, as well as the indirect effects of fear of COVID-19 were obtained through structural equation modeling using IBM SPSS Amos (version 26, IBM Corp., Armonk, NY, USA). Bootstrapped confidence intervals (95%) and ***p***-values were used to assess the significance of effects.

Moderation analyses was conducted using the PROCESS macro [29] in SPSS. The variables that were used to create the interaction terms were mean-centered. To plot the nature of significant moderating effects, three focal points—one SD above the mean, the mean, and one SD below the mean—were used to create groups.

## 3. Results

The descriptive statistics (means and standard deviations), reliabilities (alpha and omega) and intercorrelations between variables are presented in Table 1. Some authors have expressed concerns that the alpha coefficient may underestimate true reliability in multi-item measurement scales [30,31], and thus both alpha and omega were calculated. With the exception of the manageability and meaningfulness subscales, the reliabilities of all the scales can be considered satisfactory (α = 0.69–0.94; ω = 0.71–0.94). The comprehensibility subscale had moderate reliability in terms of alpha (α = 0.69) but satisfactory reliability in terms of omega (ω = 0.71). The reliability of the manageability subscale was moderate (α = 0.59, ω = 0.60), while the reliability of the meaningfulness subscale can be regarded as low α = 0.52, ω = 0.53).

In terms of intercorrelations, fear of COVID-19 was negatively related to the dimensions of SOC (comprehensibility: r = −0.16, *p* = 0.002; manageability: r = −0.23, *p* < 0.001; meaningfulness: r = −0.14, *p* = 0.010) and positively related to both emotional exhaustion (r = 0.26, *p* < 0.001) and depersonalization (r = 0.24, *p* < 0.001). Burnout was negatively related to emotional exhaustion (comprehensibility: r = −0.49, *p* < 0.001; manageability: r = −0.45, *p* < 0.001; meaningfulness: r = −0.45, *p* < 0.001) and depersonalization (comprehensibility: r = −0.44, *p* < 0.001; manageability: r = −0.48, *p* < 0.001; meaningfulness: r = −0.45, *p* < 0.001) as well as positively related to personal accomplishment (comprehensibility: r = 0.36, *p* < 0.001; manageability: r = 0.35, *p* < 0.001; meaningfulness: r = 0.50, *p* < 0.001).

**Figure 1:**
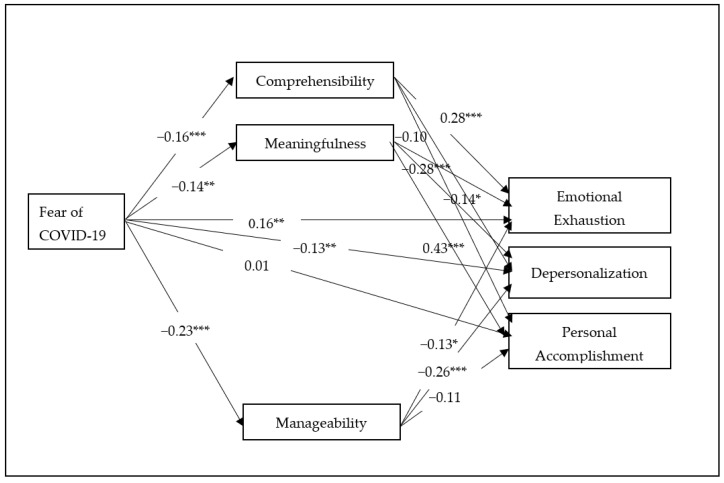
Structural equation model of the interrelationship between variables. Note. Regression weights are standardized *** *p* < 0.001, ** *p* < 0.01, * *p* < 0.05.

The structural equation model that was used to examine the direct and indirect effects of fear of COVID-19 is presented in Figure 1. In this model, fear of COVID-19 was considered the predictor variable with the indices of burnout as the outcome variables. The three dimensions of burnout were regarded as mediator variables.

The direct effects resulting from the structural equation model in Figure 1 are presented in Table 2. Except for the relationship between comprehensibility and personal accomplishment, as well as the relationship between manageability and personal accomplishment, all of the hypotheses were supported.

In particular, Table 2 reveals, with regard to the hypotheses, that:

**Hypothesis** **1** **(H1).** *Comprehensibility was negatively associated with emotional exhaustion (β = −0.281, CI95 [−0.391, −0.170], p < 0.001).*

**Hypothesis** **2** **(H2).** *Comprehensibility was negatively associated with depersonalization (β = −0.142, CI95 [−0.248, −0.031], p = 0.028).*

**Hypothesis** **4** **(H4).** *Manageability was negatively associated with emotional exhaustion (β = −0.129, CI95 [−0.243, −0.024], p = 0.048).*

**Hypothesis** **5** **(H5).** *Manageability was negatively associated with depersonalization (β = −0.256, CI95 [−0.360, −0.154], p < 0.001).*

**Hypothesis** **7** **(H7).** *Meaningfulness was negatively associated with emotional exhaustion (β = −0.282, CI95 [−0.380, −0.188], p < 0.001).*

**Hypothesis** **8** **(H8).** *Meaningfulness was negatively associated with depersonalization (β = −0.295, CI95 [−0.378, −0.209], p < 0.001).*

**Hypothesis** **9** **(H9).** *Meaningfulness was positively associated with personal accomplishment (β = 0.434, CI95 [0.345, 0.521], p < 0.001).*

The indirect effects of fear of COVID-19 on indices of burnout are reported in Table 3.

Table 3 indicates that, with the exception of the mediating role of comprehensibility and manageability in the relationship between fear of COVID-19 and personal accomplishment, all of the hypotheses were supported:

**Hypothesis** **10** **(H10).** *Comprehensibility mediated the relationship between fear of COVID-19 and emotional exhaustion (β = 0.046, CI95 [0.035, 0.130], p < 0.001).*

**Hypothesis** **11** **(H11).** *Manageability mediated the relationship between fear of COVID-19 and emotional exhaustion (β = 0.030, CI95 [0.011, 0.034], p = 0.034).*

**Hypothesis** **12** **(H12).** *Meaningfulness mediated the relationship between fear of COVID-19 and emotional exhaustion (β = 0.039, CI95 [0.022, 0.105], p = 0.005).*

**Hypothesis** **13** **(H13).** *Comprehensibility mediated the relationship between fear of COVID-19 and depersonalization (β = 0.023, CI95 [0.009, 0.072], p = 0.013).*

**Hypothesis** **14** **(H14).** *Manageability mediated the relationship between fear of COVID-19 and depersonalization (β = 0.059, CI95 [0.045, 0.134], p < 0.001).*

**Hypothesis** **15** **(H15).** *Meaningfulness mediated the relationship between fear of COVID-19 and depersonalization (β = 0.041, CI95 [0.022, 0.094], p = 0.005).*

**Hypothesis** **18** **(H18).** *Meaningfulness mediated the relationship between fear of COVID-19 and personal accomplishment (β = −0.060, CI95 [−0.092, −0.021], p = 0.006).*

Since comprehensibility and manageability did not demonstrate a mediating effect with respect to the relationship between fear of COVID-19 and personal accomplishment, the potential moderating role of these dimensions was examined using moderation analyses. The results of these analyses are presented in Table 4.

**Figure 2:**
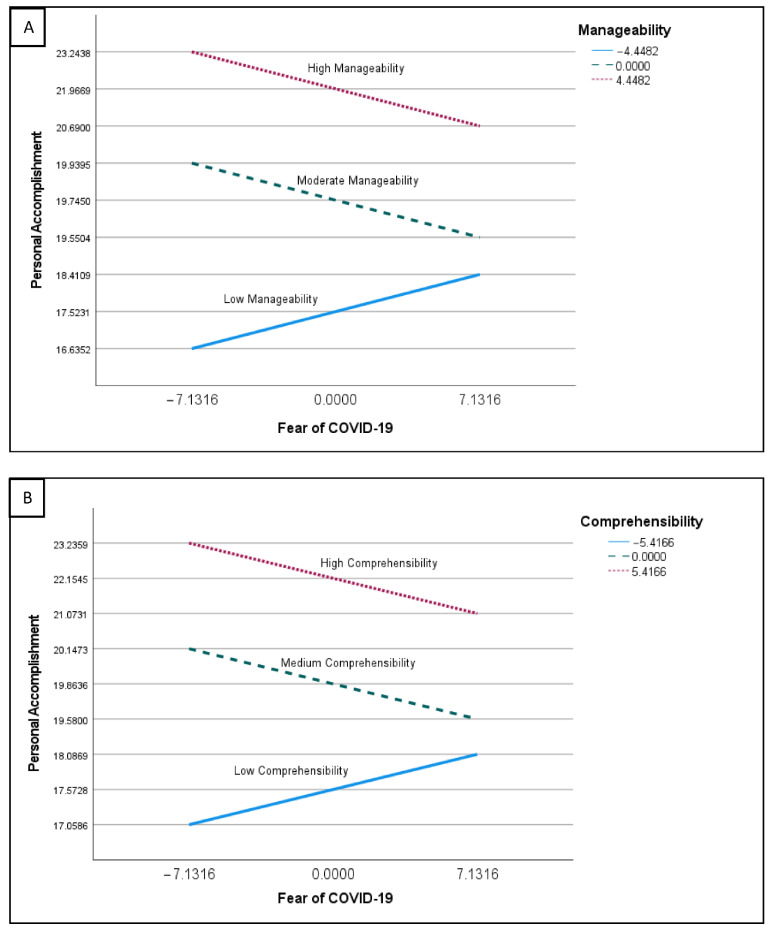
The relationship between fear of COVID-19 and personal accomplishment for three different groups in terms of manageability and comprehensibility. Note. Manageability: −4.45 = low (solid line), 0.00 = moderate (dash line), 4.45 = high (dotted line). Comprehensibility: −5.42 = low (solid line), 0.00 = moderate (dash line), 5.42 = high (dotted line).

Table 4 indicates that the interaction terms (fear of COVID-19 × comprehensibility and fear of COVID-19 × manageability) were significant. This indicates that comprehensibility and manageability had a moderating effect on fear of COVID-19. The exact nature of this moderating effect is illustrated in Figure 2A,B.

Figure 2A indicates that those who reported high levels of manageability reported higher levels of personal accomplishment at both high and low levels of fear of COVID-19 compared with those with moderate or low scores in manageability. Those who reported low scores in manageability reported the lowest levels of personal accomplishment in relation to fear of COVID-19. Figure 2B demonstrates the same pattern for comprehensibility, with those reporting high scores in comprehensibility reporting higher levels of personal accomplishment at high and low levels of fear of COVID-19 than those reporting moderate or low levels of comprehensibility.

## 4. Discussion

This study aimed to investigate the health-sustaining, moderating, and mediating roles of SOC in the relationship between fear of COVID-19 and burnout. There were several important findings. First, the study confirmed that all dimensions of SOC were strongly associated with the three dimensions of burnout, suggesting that SOC plays a health-sustaining role. High levels of meaningfulness, comprehensibility, and manageability were associated with low levels of emotional exhaustion and depersonalization. In addition, high levels of meaningfulness were associated with high levels of personal accomplishment. These results replicate the findings in the existing literature [32,33] regarding the relationship between SOC and mental health. Stoyanova and colleagues [16], for example, reported strong associations between the three components of SOC and the burnout dimensions among health care workers and suggested that the combination of the cognitive, motivational, and instrumental aspects of SOC can predict burnout. Schäfer and colleagues [34] investigated the potential modulatory effects of SOC on mental health before and after the COVID-19 outbreak and found that SOC buffered the impact of stressors on psychological distress.

Second, the study found that meaningfulness mediated the relationship between fear of COVID-19 and all indices of burnout. Comprehensibility and manageability only mediated the relationship between fear of COVID-19 and emotional exhaustion and depersonalization. Meaningfulness is typically associated with fulfilling relationships with significant others and, for teachers, relationships with students, parents, and colleagues have been identified as central to their job satisfaction. It is therefore probable that appraisals of the benefits derived from these relationships may have outweighed fears of COVID-19 and protected against burnout [35]. Existing studies have underscored that lack of organizational support and inadequate resources (e.g., limited access to personal protective equipment and overcrowded classrooms) contribute significantly to emotional exhaustion and depersonalization [36]. These dimensions of burnout can also be explained by Herzberg’s two-factor hygiene theory, which posits that a lack of fulfillment of basic hygiene needs (e.g., sanitary working conditions) can lead to withdrawal from work, a sense of disillusionment, and indifferent attitudes [37]. High levels of emotional exhaustion and depersonalization have been associated with greater concerns about infecting oneself and others [33].

For teachers in the current study, it is probable that considering fear of COVID-19 logical and understandable normalized fear and reduced psychological distress among teachers. Teachers may also have relied on personal resources available to them (e.g., support from colleagues, parents, and school administrators) to manage their fear of COVID-19 and remain committed to their profession. Previous studies (e.g., [35]) reported that professional values enhance feelings of commitment and motivate professionals to work harder in stressful situations. For teachers, a sense of obligation toward their students and the expectations of parents and their communities may have served as motivating factors.

Third, the study found that comprehensibility and manageability played a moderating role in the relationship between fear of COVID-19 and personal accomplishment. In other words, teachers with high levels of comprehensibility and manageability reported high levels of personal accomplishment at low and high levels of fear of COVID-19 compared with those who reported moderate or low levels of comprehensibility and manageability. These results are consistent with previous studies focusing on SOC and burnout (e.g., [36,38]). SOC reflects an orientation toward acknowledging challenges and solving problems through the effective identification and use of available resources. Thus, irrespective of the level of a stressor (i.e., fear of COVID-19), the presence of SOC can act as a protective factor.

These findings have implications for intervention. Studies examining resilience and recovery following mass trauma have confirmed that adverse mental health outcomes are not always a given. Instead, there are several pathways in negotiating the transition from crisis to health and it is imperative to identify and enhance health-promoting capacities and resources. Given the potential protective role of SOC in the relationship between fear of COVID-19 and burnout, interventions aimed at enhancing this psychological resource are vital in promoting the psychological well-being of teachers as well as other frontline workers. Interventions to build SOC need to consider individual-level factors such as training in the acquisition of skills in active problem-solving, time management, assertiveness training, and optimal communication methods. Interventions at the organizational level can include promoting structure and clear communication, as well as increasing awareness of and access to existing resources [33].

## 5. Limitations

With regard to the limitations of the study, caution is recommended in the generalization of its results, as the sample selection procedure was not randomized, and most participants were recruited from a single geographic area. Furthermore, most participants were female, representing an unequal distribution and potentially introducing certain biases. Finally, the study used an electronic survey, and it is likely that only participants with access to the Internet and interest in the topic responded.

## 6. Conclusions

This study investigated the health-sustaining, moderating, and mediating roles of SOC in the relationship between fear of COVID-19 and burnout in a sample of South African school teachers. The dimensions of SOC were found to be the mechanism through which fear of COVID-19 impacts burnout. When fear of COVID-19 is experienced as comprehensible, meaningful, and manageable, its impact on burnout can be reduced. Therefore, interventions aimed at enhancing SOC may be particularly beneficial toward supporting teachers in coping with the pandemic.

## Figures and Tables

**Table 1:** Descriptive statistics, reliabilities, and intercorrelations between variables.

	1	2	3	4	5	6	7
1. Fear of COVID-19	-						
2. Comprehensibility	−0.16 **	-					
3. Manageability	−0.23 ***	0.71 ***	-				
4. Meaningfulness	−0.14 **	−0.43 ***	0.42 ***	-			
5. EE	0.26 ***	−0.49 ***	−0.45 ***	−0.45 ***	-		
6. DP	0.24 ***	−0.44 ***	−0.48 ***	−0.45 ***	0.71 ***	-	
7. PA	−0.09	−0.35 ***	0.35 ***	0.50 ***	−0.31 ***	0.34 ***	-
Mean	20.9	20.8	15.3	19.8	25.0	15.2	20.0
SD	7.1	5.4	4.4	4.1	7.5	7.4	6.9
Alpha	0.91	0.69	0.59	0.52	0.94	0.85	0.84
Omega	0.91	0.71	0.60	0.53	0.94	0.86	0.84

Note. EE = emotional exhaustion, DP = depersonalization, PA = personal accomplishment. *** *p* < 0.001, ** *p* < 0.01.

**Table 2:** The direct effects of fear of COVID-19 and sense of coherence on burnout.

Direct Effect	Beta	SE	β	95% CI	*p*
Fear of COVID-19→EE	0.257	0.081	0.164	[0.085, 0.248]	0.003
Fear of COVID-19→DP	0.173	0.066	0.128	[0.049, 0.203]	0.010
Fear of COVID-19→PA	0.010	0.045	0.011	[−0.068, 0.095]	0.790
Comprehensibility→EE	−0.581	0.139	−0.281	[−0.391, −0.170]	0.001
Comprehensibility→DP	−0.251	0.113	−0.142	[−0.248, −0.031]	0.028
Comprehensibility→PA	0.112	0.097	0.095	[−0.030, 0.229]	0.221
Manageability→EE	−0.325	0.167	−0.129	[−0.243, −0.024]	0.048
Manageability→DP	−0.552	0.137	−0.256	[−0.360, −0.154]	0.001
Manageability→PA	0.169	0.108	0.114	[−0.006, 0.233]	0.118
Meaningfulness→EE	−0.765	0.156	−0.282	[−0.380, −0.188]	0.001
Meaningfulness→DP	−0.687	0.124	−0.295	[−0.378, −0.209]	0.001
Meaningfulness→PA	0.694	0.095	0.434	[0.345, 0.521]	0.001

Note. Beta = unstandardized coefficient, β = standardized coefficient. CI = confidence interval. EE = emotional exhaustion, DP = depersonalization, PA = personal accomplishment.

**Table 3:** The indirect effects of fear of COVID-19 on indices of burnout.

Indirect Effect	Beta	SE	β	95% CI	*p*
Fear of COVID-19→Comprehensibility→EE	0.072	0.028	0.046	[0.035, 0.130]	0.000
Fear of COVID-19→Comprehensibility→DP	0.031	0.018	0.023	[0.009, 0.072]	0.013
Fear of COVID-19→Comprehensibility→PA	−0.014	0.028	−0.016	[−0.042, 0.002]	0.160
Fear of COVID-19→Manageability→EE	0.047	0.027	0.030	[0.011, 0.034]	0.034
Fear of COVID-19→Manageability→DP	0.080	0.026	0.059	[0.045, 0.134]	0.000
Fear of COVID-19→Manageability→PA	−0.024	0.017	−0.026	[−.056, −0.001]	0.093
Fear of COVID-19→Meaningfulness→EE	0.061	0.026	0.039	[0.022, 0.105]	0.005
Fear of COVID-19→Meaningfulness→DP	0.055	0.022	0.041	[0.022, 0.094]	0.005
Fear of COVID-19→Meaningfulness→PA	−0.055	0.021	−0.060	[−0.092, −0.021]	0.006

Note. EE = emotional exhaustion, DP = depersonalization, PA = personal accomplishment.

**Table 4:** Moderation analysis of the role that comprehensibility and manageability play in the relationship between fear of COVID-19 and personal accomplishment.

Variable	Beta	SE	95% CI	*p*
Manageability as moderator				
Fear of COVID-19	−0.027	0.050	[−0.125, 0.070]	0.582
Manageability	0.500	0.079	[0.344, 0.656]	0.000
Fear of COVID 19 × Manageability	−0.034	0.011	[−0.056, −0.012]	0.003
Comprehensibility as moderator				
Fear of COVID-19	−0.040	0.049	[−0.136, 0.056]	0.416
Comprehensibility	0.423	0.064	[0.298, 0.549]	0.000
Fear of COVID 19 × Comprehensibility	−0.021	0.009	[−0.039, −0.003]	0.026

## Data Availability

The data sets generated and/or analyzed during the current study are available from the corresponding author upon reasonable request.
